# Developmental Ethanol Exposure Causes Reduced Feeding and Reveals a Critical Role for Neuropeptide F in Survival

**DOI:** 10.3389/fphys.2018.00237

**Published:** 2018-03-22

**Authors:** Amanda Guevara, Hillary Gates, Brianna Urbina, Rachael French

**Affiliations:** Biological Sciences, San Jose State University, San Jose, CA, United States

**Keywords:** Neuropeptide Y, feeding behavior, *Drosophila melanogaster*, developmental ethanol exposure, developmental lethality

## Abstract

Food intake is necessary for survival, and natural reward circuitry has evolved to help ensure that animals ingest sufficient food to maintain development, growth, and survival. Drugs of abuse, including alcohol, co-opt the natural reward circuitry in the brain, and this is a major factor in the reinforcement of drug behaviors leading to addiction. At the junction of these two aspects of reward are alterations in feeding behavior due to alcohol consumption. In particular, developmental alcohol exposure (DAE) results in a collection of physical and neurobehavioral disorders collectively referred to as Fetal Alcohol Spectrum Disorder (FASD). The deleterious effects of DAE include intellectual disabilities and other neurobehavioral changes, including altered feeding behaviors. Here we use *Drosophila melanogaster* as a genetic model organism to study the effects of DAE on feeding behavior and the expression and function of Neuropeptide F. We show that addition of a defined concentration of ethanol to food leads to reduced feeding at all stages of development. Further, genetic conditions that reduce or eliminate NPF signaling combine with ethanol exposure to further reduce feeding, and the distribution of NPF is altered in the brains of ethanol-supplemented larvae. Most strikingly, we find that the vast majority of flies with a null mutation in the NPF receptor die early in larval development when reared in ethanol, and provide evidence that this lethality is due to voluntary starvation. Collectively, we find a critical role for NPF signaling in protecting against altered feeding behavior induced by developmental ethanol exposure.

## Introduction

Pediatricians often tell parents that their child won't starve themselves to death, and will eat when hungry. This is largely true: feeding behavior in both invertebrates and vertebrates is driven by two factors: hunger (induced by reduced energy availability) and food reward, and these two factors combine to ensure that animals consume sufficient food to allow further growth and survival. Nevertheless, there are developmental conditions that reduce the ability or willingness of children to eat. One such condition is Fetal Alcohol Spectrum Disorder (FASD), a collection of neurobehavioral and physical abnormalities that are a result of developmental alcohol exposure (DAE) (Jones and Smith, 1973; Kvigne et al., 2004; Dörrie et al., 2014). Feeding abnormalities, including anorexia and dysphagia, are commonly associated with FASD (Clarren and Smith, 1978), and feeding anomalies associated with chronic ethanol exposure have previously been characterized in adult mammals (Štrbák et al., 1998). However, despite the growing body of research on chronic ethanol exposure and feeding, investigations into changes in feeding behavior after DAE are nearly non-existent.

Both hunger and food reward appear to be regulated by the appetite-stimulating molecule Neuropeptide Y (NPY), a 36-amino acid neuropeptide that signals through a variety of G-protein-coupled receptors (GPCRs) (Clark et al., 1984; Segal-Lieberman et al., 2002). Injections of NPY into the hypothalamus of rats induce feeding (Clark et al., 1984), while NPY ablation in mice results in an impaired refeeding response after fasting (Segal-Lieberman et al., 2002). Hypothalamic NPY expression is increased by fasting, an effect that is reversed by refeeding (reviewed in Heilig et al., 1994). Neuropeptide F (NPF), the sole *Drosophila* ortholog of NPY (Brown et al., 1999), signals through NPFR1, a GPCR related to mammalian NPY receptors. Like NPY, NPF regulates feeding behavior. In flies, NPF is expressed in six neurons in the third instar larval central nervous system: two pairs in the medial and lateral protocerebrum, and one pair in the subesophageal ganglion (SEG). Expression in the adult brain is more widespread (Brown et al., 1999; Lee et al., 2006). Reduced NPF signaling causes decreased feeding in larvae, and changes in NPF expression regulate developmental changes in foraging behavior, with high levels of NPF driving foraging in younger larvae (Wu et al., 2003).

NPY/NPF is implicated in the regulation of both natural rewards, such as food and sex, as well as drug rewards. Food containing a high concentration of sugar (20%) increases both NPF mRNA expression and NPF release in larvae (Shen and Cai, 2001), and overexpression of the fly NPF receptor (NPFR1) is sufficient to induce well-fed larvae to consume noxious food, while silencing of NPFR1 neurons reduces consumption of noxious food in food-deprived larvae (Wu et al., 2005).

Altered NPY/NPF signaling also results in changes in ethanol-induced behaviors. For example, in mice, knocking out NPY or its receptor NPY-Y1 leads to decreased ethanol sensitivity as measured by time to return to normal posture after an intraperitoneal inebriating dose of ethanol (loss of righting reflex). NPY knockout mice were able to right themselves significantly faster than control mice. In addition, mice deficient in NPY signaling show increased ethanol consumption compared to wildtype animals (Thiele et al., 1998, 2002). Similarly, flies with a loss of function in *npf* or *npfr1* display decreased ethanol sensitivity, as measured by the time it takes animals to become immobile when exposed to a sedating concentration of ethanol vapor (Wen et al., 2005). Finally, in sexually deprived male flies there is a decrease in NPF expression and a concomitant increase in ethanol consumption, while activation of NPF neurons reduces ethanol reward, as measured by the preference of animals for ethanol-containing food over food without ethanol (Shohat-Ophir et al., 2012).

In the wild, female *Drosophila* preferentially deposit their eggs in rotting fruit, resulting in larval exposure to concentrations of ethanol ranging from 6 to 11%, much higher than those usually tolerated by insects, and this is due in part to high levels of expression of the alcohol-detoxifying enzyme alcohol dehydrogenase (Gibson et al., 1981; McKechnie and Morgan, 1982). Ethanol at these concentrations is nonetheless toxic to developing *Drosophila* larvae, leading to decreased cell division, slow growth, and, sometimes, to the deaths of at least 50% of the flies (McClure et al., 2011).

Several hypotheses have been proposed to explain the preference of flies for egg deposition sites with high ethanol concentrations. At low concentrations, ethanol is beneficial to fly development (Parsons et al., 1979); thus the consumption of toxic levels may be merely a consequence of selection for preference of lower, healthful ethanol concentrations. Alternatively, as ethanol is also toxic to many of the organisms that prey on developing fly larvae and well as other insects with which the larvae compete for resources, ethanol preference may have evolved as a way to utilize an environment that competitors and parasites find intolerable (Milan et al., 2012).

Here we use our previously-established *Drosophila* model for DAE (McClure et al., 2011; Logan-Garbisch et al., 2014) to examine the effects of DAE on feeding behavior and investigate the hypothesis that DAE leads to reduced hunger or food reward. We show that ethanol-supplemented flies consistently eat less than control animals, at every stage of development. Additionally, we find that NPF expression is increased in the brains of ethanol-supplemented larvae, and loss of NPF signaling enhances ethanol-induced anorexia. Finally, we show that while loss of NPF signaling normally has no effect on survival, loss of function of the NPF receptor (NPFR1) combined with rearing in ethanol-supplemented food results in early larval lethality, and provide evidence that this lethality is due to decreased food intake. Our data raise the possibility that NPF signaling during larval development is an adaptation that helps to allow *Drosophila* larvae to exploit environments with a high concentration of ethanol.

## Materials and methods

### Fly stocks, genetics, and husbandry

Fly stocks were maintained at 25°C on standard corn meal and molasses medium. Fly strains were obtained from the Bloomington *Drosophila* Stock Center (Bloomington, Indiana) and the strains used were: *w*^*1118*^*; PBac{PB}npfr*^*c01896*^ (Bloomington Stock #10747), *w*^*1118*^*; da-GAL4* (Bloomington Stock #12429), and *UAS-npf*^*RNAi*^ (Bloomington Stock #27237). For the *npf* RNAi experiments, *da-GAL4/da-GAL4* virgin females were crossed with *UAS-npf*^*RNAi*^*/UAS-npf*^*RNAi*^ males. Background controls for RNAi experiments were generated by crossing *da-GAL4/da-GAL4* virgin females to males from our standard laboratory stock strain (*w*^*1118*^*, Wild-Type Berlin (w:WTB))*, or *UAS- UAS-npf*^*RNAi*^*/UAS-npf*^*RNAi*^ males to *w; WTB* virgin females. The *npfr1*^*c01896*^ mutation behaves as a genetic null allele; Lee and colleagues found that the electrophysiological phenotypes of flies homozygous for the *npfr1*^*c01896*^ mutation were indistinguishable from flies transheterozygous for *npfr1*^*c01896*^ and a deletion uncovering *npfr1* (Lee et al., 2017).

Throughout the manuscript, “food” refers to fly food prepared according to the Bloomington Stock Center's Cornmeal, Molasses and Yeast Recipe (https://bdsc.indiana.edu/information/recipes/molassesfood.html), with additions as described in the text.

### Ethanol rearing

Eggs were collected on 35 mm Petri dishes containing standard fly food. One hundred eggs (per vial) were transferred to vials containing fly food with 7% ethanol or no ethanol (control). For ethanol-containing food, food is allowed to cool to 70–75°C, at which point ethanol is added to the appropriate concentration (and the same volume of water is added to control food). Vials are transferred to a closed 40-cup food storage container (Rubbermaid Home Products, Fairlawn, OH) filled with 1 L of 7% ethanol (experimental conditions) or deionized water (control conditions). The ethanol bath ensures that animals are exposed to ethanol throughout development, which continues for another 10–16 days. Newly eclosed adult flies were counted and collected daily and kept at 25°C (~12 h light, ~12 h dark), and these data were used to calculate the percentage of flies that survived to adulthood.

To determine critical periods for the deaths of NPF signaling mutants on ethanol-containing food, larvae were collected from control food plates as they reached the desired developmental stage (first, second, or third larval instar), transferred to 7%-ethanol-containing food (or control food) and grown as described above. The total number of pupae was counted for each vial, and used to calculate: (1) The percentage of larvae that survived to pupation, and (2) the percentage of pupae that survived to adulthood.

### Feeding assays

**Figure 1:**
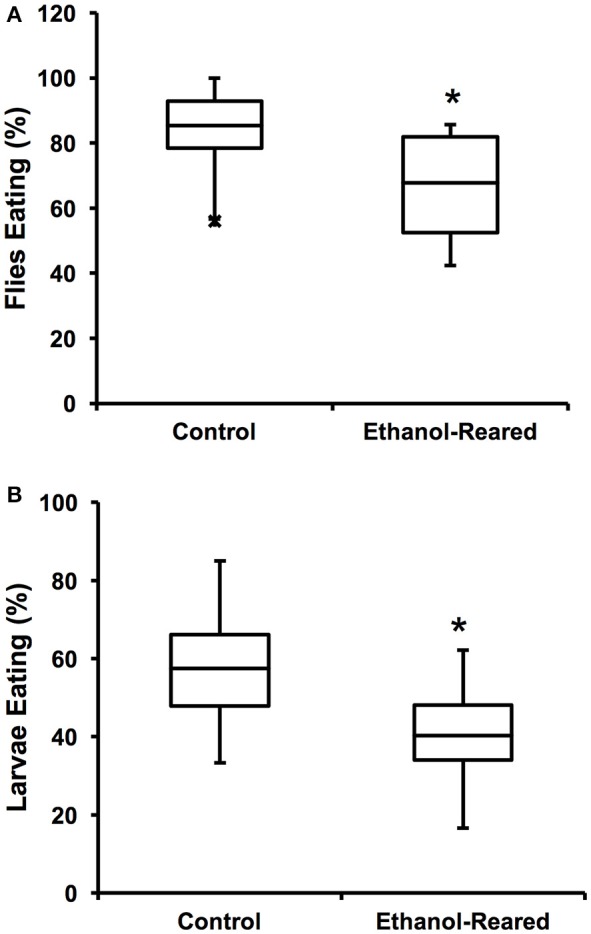
Ethanol-rearing results in reduced feeding. **(A)** Percentage of adult female flies that ate within a 3-min interval after 6 h of food deprivation. (*N* = 12 for control, 14 for ethanol-reared. *P* = 0.0056, Student's *t*-Test). **(B)** Percentage of early third instar larvae that ate within a 20-min interval after 2 h of food deprivation (*N* = 7, *P* = 0.035, Student's *t*-Test). Center lines show the sample mean; box limits indicate the 25th and 75th percentiles as determined by R software; whiskers extend 1.5 times the interquartile range from the 25th and 75th percentiles; outliers are represented by “x.” ^*^P < 0.05.

**Figure 2:**
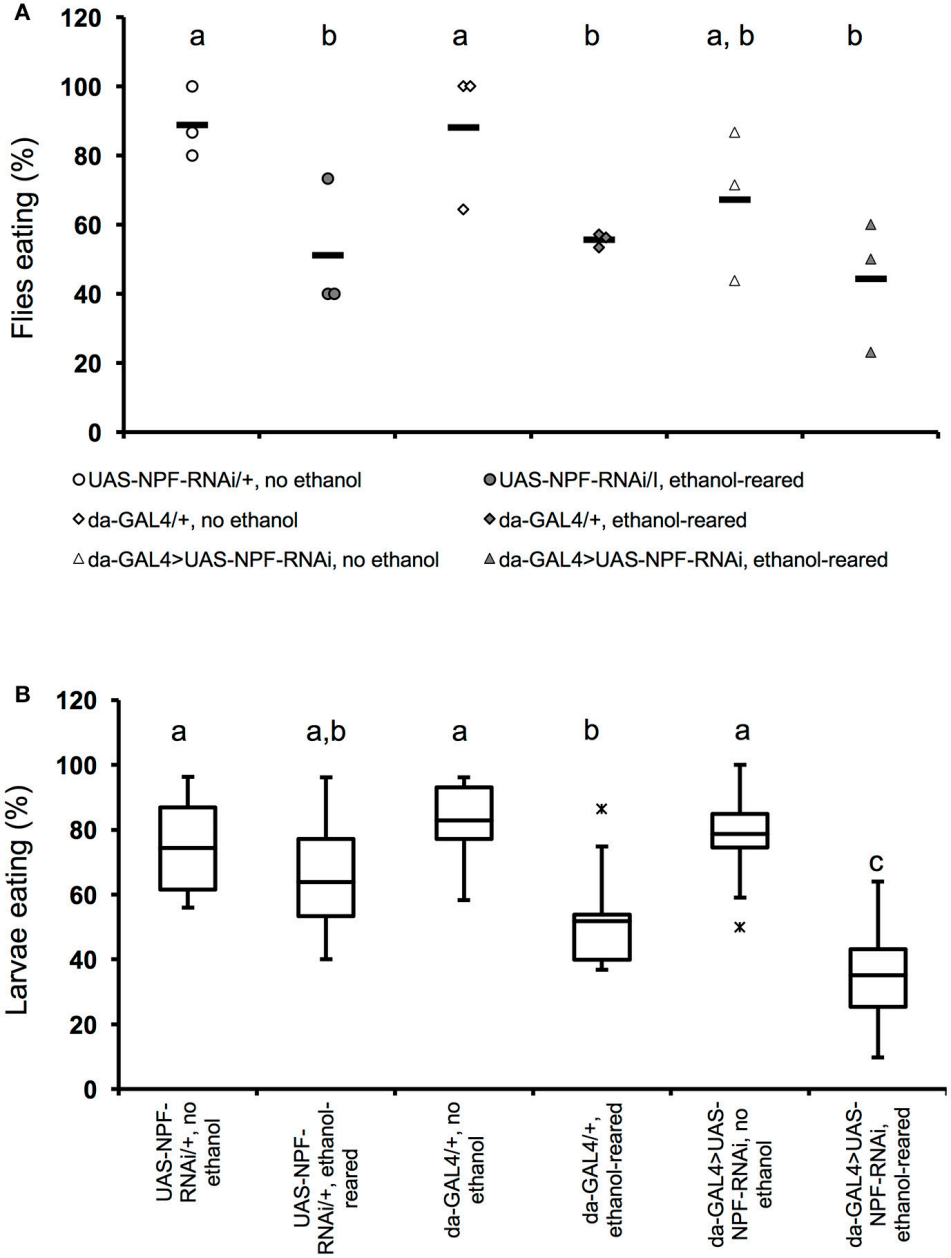
Flies with partial loss of *npf* signaling show reduced feeding when ethanol-supplemented. **(A)** Percentage of adult female flies that ate within a 4-min interval after 6 h of food deprivation. (*N* = 3 for all conditions and genotypes, *P* = 0.0022 for the effect of ethanol-rearing, NS for the effect of loss of *npf* (*P* = 0.24, two-way ANOVA with Tukey's *post-hoc* analysis). Solid bars indicate means; individual data points are represented as open or closed circles. **(B)** Percentage of early third instar larvae that ate within a 20-min interval after 2 h of food deprivation (*N* = 11, 11, 8 6, 10, 8. *P* < 0.0001 for the effect of ethanol-rearing, *P* = 0.11 for the effect of loss of *npf*, *P* = 0.0053 for the interaction between genotype and condition, two-way ANOVA with Tukey's *post-hoc* analysis). Center lines show the sample mean; box limits indicate the 25th and 75th percentiles as determined by R software; whiskers extend 1.5 times the interquartile range from the 25th and 75th percentiles; outliers are represented by “x.” Boxes sharing the same letter do not differ significantly, while boxes with different letters are significantly different (*P* < 0.05).

Adult feeding assays were conducted on mated females. Flies analyzed for behavior were aged 2–5 days after eclosion and anesthetized briefly with CO_2_ (<5 min) no less than 24 h before feeding assays. To measure feeding motivation, mated females were collected and kept food-deprived in vials with a 25 mm-diameter circle of water-saturated Whatman Grade 1 filter paper for 6 h prior to feeding. Twenty-five flies were allowed to feed on food mixed with 0.5% v/v of FD&C Blue Dye #1, and confirmation of food consumption was performed by visual assay for the presence of blue dye in the gut. Motivation was calculated as the proportion of flies that had eaten within 3–4 min of the start of the assay. For Figure 1A, we tested 12 control and 14 ethanol-reared groups of 25 flies each. For Figure 2A, we tested 3 groups of 25 flies for each combination of condition and genotype.

**Figure 3:**
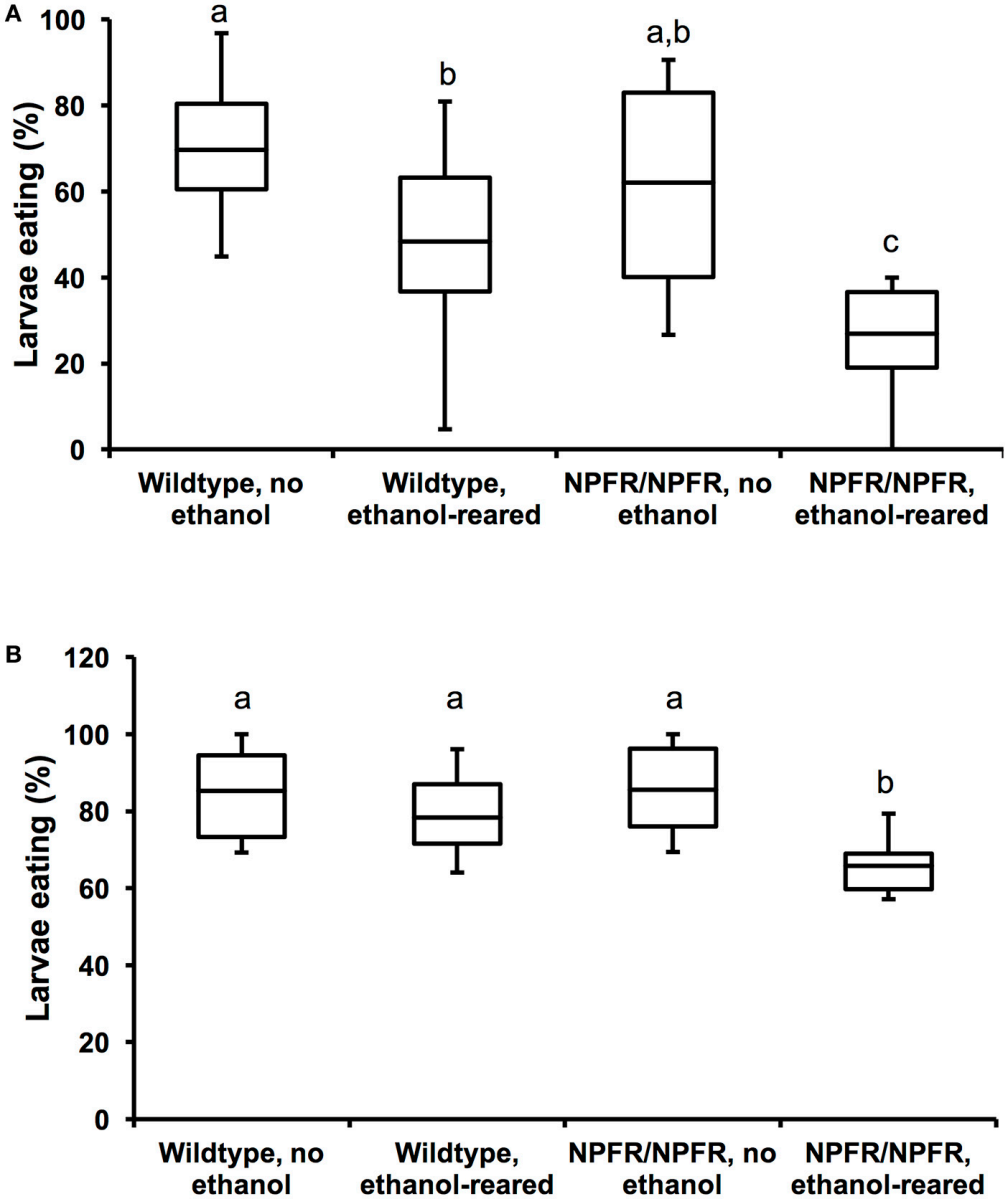
Loss of NPF signaling enhances feeding deficits in ethanol-supplemented flies**. (A)** Percentage of unstarved first instar larvae that ate during a 20-min interval. Both ethanol-rearing and loss of *npfr1* result in reduced feeding (*N* = 12, 11, 11, 11, *P* < 0.001 for the effect of ethanol-rearing, *P* = 0.009 for the effect of mutation of *npfr1*, two-way ANOVA with Tukey's *post-hoc* analysis). **(B)** Percentage of unstarved first instar larvae that ate during a 45-min interval. (*N* = 10, 10, 12, 10, *P* = 0.003 for the effect of ethanol-rearing, *P* = 0.13 for the effect of *npfr1* mutation, *P* = 0.046 for the interaction between ethanol and genotype, two-way ANOVA with Tukey's *post-hoc* analysis). Center lines show the sample mean; box limits indicate the 25th and 75th percentiles as determined by R software; whiskers extend 1.5 times the interquartile range from the 25th and 75th percentiles. Boxes sharing the same letter do not differ significantly, while boxes with different letters are significantly different (*P* < 0.05).

For larval feeding assays, first instar or young third instar larvae were collected at 16 or approximately 72 h after egg-laying (AEL), respectively. Third instar larvae were kept food-deprived for 2 h prior to feeding, while first instar larvae were not starved. 30 larvae were placed onto 3% agarose plates and allowed to feed on yeast paste containing 0.5% v/v FD&C Blue Dye #1 for 20 min. A larva was considered to have eaten by the presence of blue dye in 3/4 its length. This measurement is a slight modification of the protocol published by Wu et al. (2005), with an increased length that helps ensure accuracy in scoring. For Figure 1B, we tested 7 control and 7 ethanol-reared groups of 30 larvae each. For Figure 2B, we tested 6–11 groups of 30 larvae for each combination of condition and genotype. For Figures 3A,B, we tested 10–12 groups of 30 larvae for each combination of condition and genotype.

### Locomotion assay

To measure locomotion, first instar larvae were transferred to a pre-marked spot on a 3% agarose plate. Larvae were allowed to move for 3 min, then their final position was marked on the plate using the point of a needle. Total distance traveled was measured as the distance of the direction connection from the starting point to the end point, and reported in mm.

### Immunostaining and imaging

Larvae were dissected in PBS + 0.3% Triton X-100 and tissues were fixed in 4% paraformaldehyde for 30 min. Tissues were then washed with 1x PBST and incubated for 2 days in a 1:750 dilution of rabbit anti-NPF (Ray Biotech, Norcross, GA) in 1X PBST. Secondary antibodies (Alexa Fluor 488 goat anti-rabbit, Jackson ImmunoResearch, Burlington, Ontario) were diluted 1:750 in 1X PBST plus 5% normal goat serum. Stained samples were mounted in Vectashield anti-fade mounting medium for imaging and all images were collected on a Zeiss LSM 700 confocal microscope using a 20X objective.

Confocal images were quantitated using NIH Image J. For pixel area, thresholded pixels were counted for each image (threshold set to 90). For total fluorescence, the integrated density of pixels for entire images was measured. Images were then calibrated for background by calculating the average mean fluorescence of four circular regions of each image, multiplying that average by the total pixel area of the image, and subtracting that number from the integrated pixel density of the image.

### Statistical analyses

All samples were tested for normality using the Shapiro-Wilk normality test (Shapiro and Wilk, 1965). Data that were non-normal were log-transformed, and statistical analyses conducted on log-transformed data (Supplemental Figure 1). All statistical analyses were conducted using two-way ANOVA with a Tukey HSD *post-hoc* or Student's *T*-test unless otherwise indicated.

## Results

### Ethanol-rearing results in reduced feeding

In order to assess the effects of DAE on feeding, we measured the flie's motivation to feed, as defined as the probability that a fly will choose to consume food within a specified time frame. To measure motivation, 25 adult female flies were starved for 6 h, then introduced into vials containing standard fly food mixed with blue dye and allowed to feed for 3 min. Feeding was assessed by the presence of blue color in the gut. Within 3 min of being transferred to blue food, 85 ± 3.6% of control animals contained food in 3/4 the length of the gut, compared with 68 ± 4.4% of ethanol-supplemented flies (Figure 1A, *N* = 12–14, *P* = 0.0056, Student's *t*-Test). These results demonstrate that DAE leads to a reduction of food present in the gut, a result similar to the reported effects of fetal alcohol exposure in humans and rodent models.

Next, we asked whether DAE also reduces larval feeding. We tested the feeding motivation of early third instar larvae (at this stage of development, larvae are still actively eating). Our results were similar to those seen with adult flies: over the course of 20 min, 57.5 ± 6.5% of control larvae fed, compared with 40.3 ± 5.8% of ethanol-supplemented larvae (Figure 1B, *N* = 7, *P* = 0.035, Student's *t*-Test). As with the adult flies, these results demonstrate that the guts of ethanol-supplemented animals contain less food than those of control animals.

### Ethanol-induced changes in feeding are mediated by Neuropeptide F

In addition to being a known “hunger” signal, Neuropeptide F (NPF) has been implicated as a regulator of response to acute ethanol exposure (Wen et al., 2005). Specifically, flies with a partial loss of function in *npf* or *npfr1*, the gene encoding the NPF receptor, displayed resistance to ethanol-induced sedation after being exposed to ethanol vapor, whereas overexpression of *npf* resulted in increased ethanol sensitivity. Since both food and alcohol activate reward pathways (Devineni and Heberlein, 2013), and ethanol-supplemented wild-type flies eat less following starvation, we hypothesized that starved flies with decreased NPF signaling would eat less compared to genotypic controls after being reared in ethanol-supplemented food.

To test this hypothesis we used the ubiquitously-expressed GAL4 line *da-GAL4* to drive expression of a double-stranded RNA interference construct targeting *npf* (*UAS-npf*^*RNAi*^). As expected, in adult animals, ethanol-rearing lead to a reduction in feeding. While 88–89% of unexposed genetic background controls ate during the 4-min observation window (88.9 ± 5.9% for *UAS-npf*^*RNAi*^/+; 88.1 ± 11.9% for *da-Gal4/*+), only 51–56% of ethanol-supplemented controls ate (51.1 ± 11.1% for *UAS-npf*^*RNAi*^/+; 55.6 ±1.2% for *da-Gal4/*+). (Figure 2A, *N* = 3 for all conditions, *p* = 0.0022 for the effect of ethanol, two-way ANOVA with Tukey HSD *post-hoc* analysis). The longer feeding window (4 vs. 3 min) in this experiment reflects the fact that, at earlier time points, differences in food intake were not significantly different, unlike in wildtype animals.

Consistent with its role in feeding, reducing *npf* expression also resulted in reduced feeding. Only 67.3 ± 12.6% of unexposed *da-Gal4/*+*; UAS-npf*^*RNAi*^*/*+ fed. Finally, rearing *da-Gal4/*+*; UAS-npf*^*RNAi*^*/*+ animals in ethanol reduced feeding still further: only 44.4 ± 11% of ethanol-supplemented *da-Gal4/*+*; UAS-npf*^*RNAi*^*/*+ animals ate. (Figure 2A). The effect of genotype on feeding was not statistically significant, likely due to small sample size (*N* = 3 for all combinations, *P* = 0.24, two-way ANOVA with Tukey *post-hoc* analysis). Thus, animals with reduced NPF signaling ate less than animals with intact NPF signal transduction, and this effect may combine with the strong effect of ethanol to reduce feeding still further.

In third instar larvae, we saw similar results—ethanol rearing alone significantly reduces feeding motivation: 74–83% of unexposed genetic background controls ate during the observation window (74.3 ± 4.4% for *UAS-npf*^*RNAi*^/+; 82.8 ± 4.5% for *da-Gal4/*+), only 52–64% of ethanol-supplemented controls ate (63.8 ± 5.8% for *UAS-npf*^*RNAi*^/+; 51.9 ± 7.5% for *da-Gal4/*+). (Figure 2B, *N* = 6–11, *p* < 0.0001 for the effect of ethanol, two-way ANOVA with Tukey HSD *post-hoc* analysis). In larvae with reduced NPF, we saw no effect on feeding in the absence of ethanol (78.8 ± 4.1% of unexposed *da-Gal4/*+*; UAS-npf*^*RNAi*^*/*+ ate during the observation window), but when *da-Gal4/*+*; UAS-npf*^*RNAi*^*/*+ larvae were reared in ethanol, we saw a significant effect on feeding: only 35 ± 6.1% of animals ate during the observation period (Figure 2B). The effect of genotype on feeding alone was not statistically significant (*N* = 8–10, *p* = 0.11 for the effect of genotype, two-way ANOVA with Tukey HSD *post-hoc* analysis). However, we detected a significant interaction between genotype and ethanol, and *post-hoc* analyses determined that this interaction was due to the effect of ethanol on feeding in *da-Gal4/*+*; UAS-npf*^*RNAi*^*/*+ larvae (*P* = 0.0053 for the interaction between ethanol and genotype, two-way ANOVA with Tukey HSD *post-hoc* analysis). Thus, as with the adult experiments described above, reducing NPF signaling alone did not have a significant effect on feeding, but in this case there was a strong effect of ethanol on feeding in *da-Gal4/*+*; UAS-npf*^*RNAi*^*/*+ animals.

In order to further test for an effect of NPF signaling on feeding in ethanol-supplemented animals, we attempted to test the feeding behavior in animals homozygous for a genetically null (Lee et al., 2017) mutation in the fly NPF receptor *npfr1* (*npfr1*^*c01896*^*)*. Surprisingly, we found that we could recover very few *npfr1*^*c01896*^*/npfr1*^*c01896*^adults or third instar larvae when the flies were reared in ethanol. These results are described in detail below, and in Figure 3. As a result of this lethality, we decided to test first instar larval feeding behavior.

When unstarved, ethanol-supplemented first instar larvae (approximately 16 h post hatching) are allowed to feed on blue food for 20 min, 48.4 ± 6.5% of wildtype and 26.9 ± 3.7% of *npfr1*^*c01896*^*/npfr1*^*c01896*^ larvae eat, compared with 69.7 ± 4.2% of unexposed wildtype and 62.1% of unexposed *npfr1*^*c01896*^*/npfr1*^*c01896*^ animals (Figure 3A, *N* = 11–22, *p* < 0.0001 for the effect of ethanol, *p* = 0.009 for the effect of genotype, two-way ANOVA with Tukey HSD *post-hoc* analysis). Most strikingly, as with *da-Gal4/*+*; UAS-npf*^*RNAi*^*/*+ larvae, there is little effect of the *npfr1*^*c01896*^ mutation on feeding under control conditions, but when *npfr1*^*c01896*^*/npfr1*^*c01896*^ mutant animals are reared in ethanol, there is a dramatic reduction in feeding (Figure 3A). Thus, while individually, loss of NPF signaling and ethanol reduce feeding by 25 and 42%, respectively, the combination of the two conditions reduces feeding by 61%.

We repeated this assay for a longer feeding time (45 min), and the results were similar: 78.3 ± 3.6% of wildtype ethanol-supplemented larvae and 65.8 ± 2.5% of *npfr1*^*c01896*^*/npfr1*^*c01896*^ larvae ate, compared with 85.3 ± 3.9 and 85.5 ± 3.2% of unexposed larvae. In this experiment, we again see no effect of the *npfr1*^*c01896*^ mutation on feeding under control conditions, and ethanol-supplemented flies appeared to “catch up” over the longer observation time, such that there is no significant effect of ethanol on feeding (Figure 3B, *N* = 20–22, *p* = 0.126 for the effect of ethanol, two-way ANOVA with Tukey HSD *post-hoc* analysis).

However, there was a significant effect of genotype, as well as a significant interaction between ethanol and genotype, and this interaction is again due to the reduction in feeding by ethanol-supplemented *npfr1*^*c01896*^*/npfr1*^*c01896*^ larvae (Figure 3B, *p* = 0.003 for the effect of genotype, *p* = 0.046 for the interaction between genotype and ethanol, two-way ANOVA with Tukey HSD *post-hoc* analysis). Thus, the combination of ethanol exposure during development and loss of NPF signaling results in a greater reduction in feeding than either condition alone.

In order to test whether reduced feeding in first-instar larvae could be a result of increased ethanol-induced sedation, as animals in this assay were taken directly from ethanol-containing (or control) food for use in the assay, we measured the distance traveled in 3 min by first-instar larvae under each set of conditions (Supplemental Figure 1). This experiment showed that ethanol does not decrease movement of the animals; in fact, the only effect of ethanol was to increase the average distance traveled in wildtype ethanol-supplemented animals (*N* = 10, *p* = 0.025, two-way ANOVA with Tukey HSD *post-hoc* analysis), while there was no difference between mutant and wildtype animals, nor any effect of ethanol-rearing on the movement of mutant animals (*N* = 10 for all conditions, *p* = 0.82, two-way ANOVA with Tukey HSD *post-hoc* analysis). It should be noted, however, that animal-to-animal variability in distance traveled is large under all conditions.

### Loss of NPF signaling enhances ethanol-induced developmental lethality

**Figure 4:**
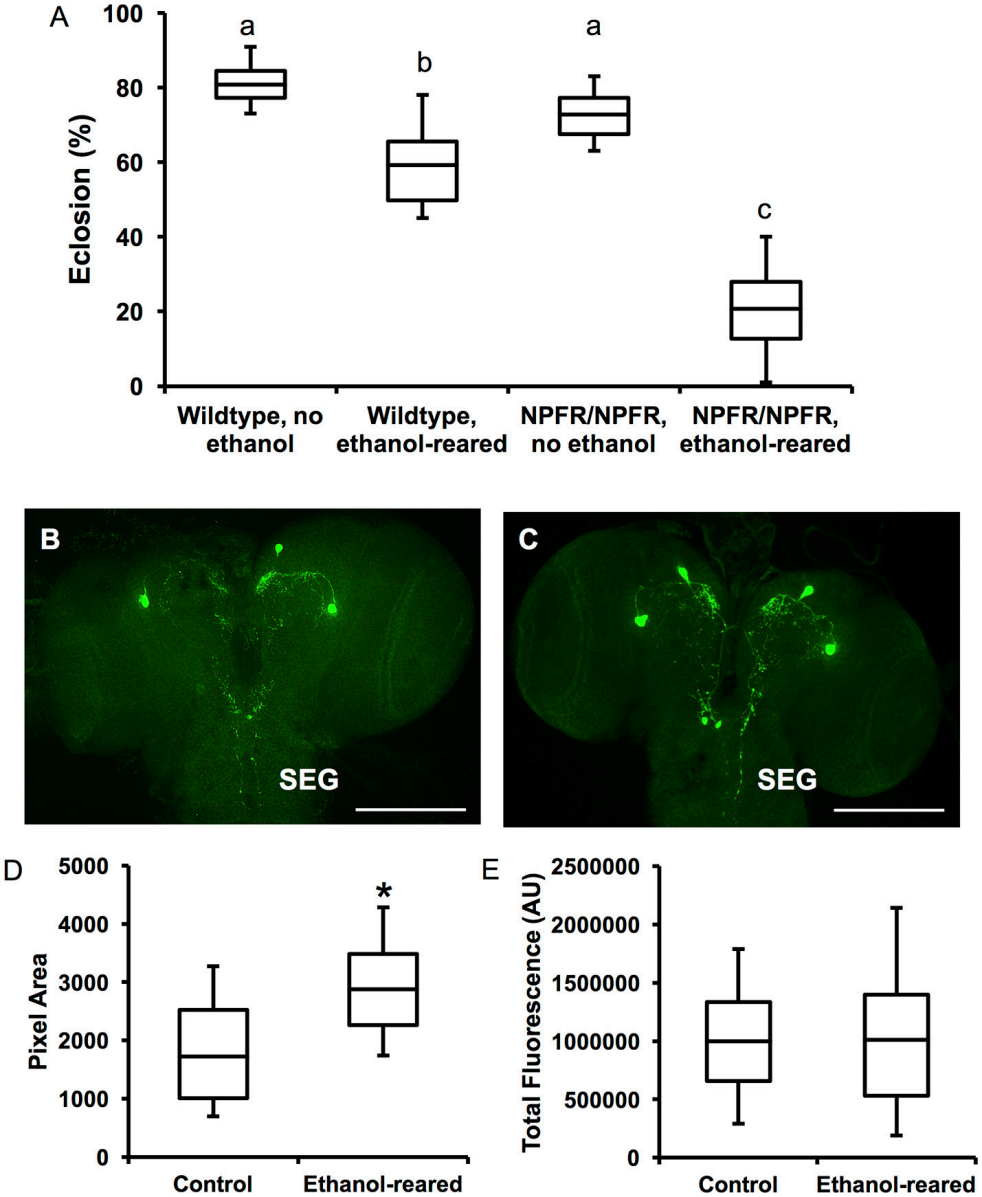
*npfr1* is necessary for survival of ethanol-supplemented flies, and developmental ethanol exposure induces a change in NPF distribution in larval brains. **(A)** Survival to adulthood of *npfr1* mutant flies reared in ethanol. (*N* = 12 for all conditions and genotypes, *P* < 0.0001 for the effect of ethanol-rearing, *P* < 0.0001 for the effect of mutation of *npfr1*, and *P* < 0.0001 for the interaction between treatment and genotype, two-way ANOVA with Tukey's *post-hoc* analysis.) Boxes sharing the same letter do not differ significantly, while boxes with different letters are significantly different (*P* < 0.05). **(B)** Confocal reconstruction of NPF-expressing neurons in the brain of a 3rd-instar larva reared in ethanol-free control food. Scale bar = 0.5 μM. **(C)** Confocal reconstruction of NPF-expressing neurons in the brain of a 3rd-instar ethanol-reared larva. Scale bar = 0.5 μM. **(D)** Quantitation of pixel area for anti-NPF fluorescence in control and ethanol-reared third-instar larval brains. (*N* = 7 brains for each condition, *P* = 0.0473, Student's *t*-Test). **(E)** Quantitation of total anti-NPF fluorescence in control and ethanol-reared third-instar larval brains. (*N* = 7 brains for each condition, *P* = 0.97, Student's *t*-Test.) Center lines show the sample mean; box limits indicate the 25th and 75th percentiles as determined by R software; whiskers extend 1.5 times the interquartile range from the 25th and 75th percentiles. ^*^P < 0.05.

Ethanol exposure during larval development leads to a reduction in survival and induces a developmental delay (McClure et al., 2011). In addition, downregulation of *npf* using *npf*-GAL4 drivers in younger larvae results in cessation of feeding and onsets of social behavior (cooperative burrowing) indicative of older third instar larvae, suggesting that NPF signaling induces changes during development (Wu et al., 2003). Finally, NPF signaling is required for adult ethanol sensitivity (Wen et al., 2005). However, to our knowledge, there is no known effect of loss of NPF signaling on survival. We were therefore surprised to discover that homozygosity for *npfr1*^*c0189*^ drastically reduces survival of ethanol-supplemented flies. 59 ± 3.3% of control flies survived to eclosion when reared in food containing 7% ethanol (*N* = 12), whereas only 21 ± 3.2% of *npfr1* mutant flies survived (*N* = 12). We found a significant interaction between genotype and condition (*N* = 48, *P* < 0.0001 for the interaction between genotype and condition, two-way ANOVA with Tukey HSD *post-hoc* analysis). Survival of *npfr1* mutant flies was no different from wildtype when reared in control food (81 ± 1.6% for wildtype; 73 ± 1.9% for *npfr1, N* = 12 for each condition, insignificant according to Tukey's HSD *post-hoc* analysis), confirming that *npfr1* is not required for survival under normal conditions (Figure 4A). These results indicate that NPF signaling is protective against ethanol-induced developmental lethality.

### Ethanol-induced lethality in *npfr1* mutant flies occurs during early larval development

Because NPF signaling is involved in food reward, and ethanol rearing causes reduced food intake, we hypothesized that animals lacking NPF signaling might not eat enough to survive. Flies eat the most during early larval development, stopping in the late third instar prior to pupation. To assess for ethanol-induced toxicity in *npfr1* mutant flies, we reared flies on food supplemented with 7% ethanol for discrete developmental periods and measured survival to pupation. The results of this analysis are presented in Table 1.

**Table 1:** Critical periods for ethanol-induced toxicity in *npfr1* mutant flies.

**Ethanol exposure**	**% Survival to pupation (± s.e.m.)**	
	**Wildtype**	***npfr1/npfr1***
No exposure	87.5(±1.9)	85.3(±2.6)
L1–L3	60.3(±2.7)	15.8(±1.7)^***^
L2–L3	71.3(±6.1)	36.0(±14.6)^*^
L3	72.5(±3.4)	76(±1.5)
M to adult	77.8(±1.9)	85.6(±7.4)

*L1, first larval instar; L2, second larval instar; L3, third larval instar, M, metamorphosis. Each rearing condition represents four vials of 100 animals/vial (n = 4). Data are presented as mean ± s.e.m*.

* *P < 0.05*,

*** *P < 0.0001, two-way ANOVA with Tukey HSD post-hoc analysis*.

In this experiment, only 15.8 ± 1.7% of *npfr1* mutant flies exposed to ethanol for the entirety of larval development pupated, compared with 60.3 ± 2.7% of wildtype flies. When the exposure period was limited to the second and third larval instars, 36 ± 14.6% of *npfr1* mutant animals pupated, while 71.3 ± 6.1% of wildtype animals began metamorphosis. However, when animals were exposed only during the third larval instar, *npfr1* mutant survival was comparable to that of controls: 76 ± 1.5% of *npfr1* mutant flies pupated, and, of those, 85.6 ± 7.4% survived to adulthood. Similarly, 72.5 ± 3.4% of wildtype animals exposed to ethanol during the third instar pupated, and, of those, 77.8 ± 1.9% survived to adulthood. Taken together, these data demonstrate that the critical period for ethanol-induced toxicity in *npfr1* mutant flies is primarily during the first and second instar larval stages, while *npfr1* mutant animals are relatively insensitive to ethanol exposure during the third instar and metamorphosis.

### Developmental ethanol exposure alters NPF expression in larval brains

Adult flies ablated of NPF/NPFR1 neurons show decreased sensitivity to ethanol-induced sedation; in addition, sexual deprivation in male flies results in both increased drinking and downregulation of NPF (Wen et al., 2005; Shohat-Ophir et al., 2012). Further, activation of NPF-expressing neurons reduces ethanol reward (Shohat-Ophir et al., 2012). Thus, NPF signaling is a likely molecular target of ethanol exposure. However, little is known about the expression of NPF in response to ethanol exposure, and nothing is known about the effects of chronic developmental ethanol exposure on NPF expression.

To examine NPF expression in ethanol-supplemented larvae, we labeled third instar larval brains with anti-NPF antibodies (Figures 4B,C). NPF is expressed in four cells in the larval protocerebrum (Brown et al., 1999), which send projections to the central brain as well as the subesophageal ganglion (SEG). The SEG contains nerves that control larval foraging and feeding (Altman and Kien, 1987) (Figures 4B,C). Staining of the cell bodies is intense in both conditions, and we see no change in this staining in the brains of ethanol-reared animals. However, we observed differences in the distribution of fluorescence in the brains of ethanol-supplemented larvae, in both the central brain and the SEG (Figures 4C,D). In particular, there is an increase of NPF-expressing neuronal projections to the SEG, very similar to the results obtained when larvae are fed on highly palatable diets (Shen and Cai, 2001). We confirmed these observations through quantitation of both fluorescence and pixel density. We find that total pixel area is significantly increased in the brains of ethanol-supplemented larvae (Figure 4D, *N* = 7 brains for each condition, *P* = 0.0473, Student's *t*-Test), while overall fluorescence is no different (Figure 4E, *N* = 7 brains for each condition, *P* = 0.97, Student's *t*-Test). These results, which indicate more overall foci of NPF fluorescence without an increase in the total amount of fluorescence, suggest that DAE alters the distribution of NPF in the larval brain, perhaps enhancing the activity of the NPF circuitry. We hypothesize that this co-opting of the natural reward circuitry by ethanol may also serve as a compensatory mechanism that normally prevents starvation of ethanol-exposed larvae by increasing feeding and foraging behavior.

## Discussion

Drugs of abuse, including alcohol, engage the natural reward systems that animals have evolved to ensure pursuit of food and sex, which are essential to the continued existence of both the individual and the species (Kelley and Berridge, 2002; Koob, 2009; Kaun et al., 2011). The neuropeptide NPY/NPF modulates both food and mating reward, and, in adult animals, NPF signaling appears to reduce both the rewarding effects and the sedative effects of ethanol (Thiele et al., 1998; Shohat-Ophir et al., 2012).

Ethanol exposure during development results in a variety of phenotypes in flies and mammals, including decreased survival, developmental delays, increased oxidative stress and changes in fat metabolism, and a variety of behavioral changes (Jones and Smith, 1973; Clarren and Smith, 1978; Kvigne et al., 2004; McClure et al., 2011; Dörrie et al., 2014; Logan-Garbisch et al., 2014). Most relevant to the current work are altered feeding behavior and responses to drugs of abuse. Children with FASD can have a variety of feeding problems (Clarren and Smith, 1978), and DAE causes reduced ethanol sedation in both flies and mammals (Middaugh and Ayers, 1988; McClure et al., 2011).

We have a well-established fly model for DAE, and here we have used it to investigate the possible effects of DAE on feeding and reward. Here, we show that ethanol-supplemented flies are less likely to eat than control animals, and that this ethanol-induced behavior change is exacerbated by loss of NPF signaling. Additionally, we show that DAE results in altered NPF signaling in larval brains. Finally, we demonstrate that ethanol-supplemented *npfr1* mutant animals die in early larval development and provide evidence that the cause of death may be starvation.

### DAE changes feeding behavior through an unknown mechanism

DAE results in reduced feeding in flies at all stages of development tested. Flies may display reduced motivation to feed for at least three reasons: they may feel less hungry, they may be behaviorally less able to find or consume food, or they may find food less rewarding and thus be less likely to eat (and eat less when they do feed). Our data do not distinguish directly between these possibilities. We are currently investigating the effects of ethanol-rearing on additional mutations that alter feeding and foraging behavior, in order to begin to address this question.

In addition, it is possible that, because ethanol is an energy-providing nutrient, animals reared in ethanol-supplemented food may find the “test” food, which lacks ethanol, to be a lower-quality food source, and stop or slow down feeding temporarily. We think this explanation is unlikely, because previous results have shown ethanol-containing food to be unpalatable (Kaun et al., 2011). However, we are unable to formally rule out such an explanation.

### DAE leads to altered distribution of NPF in larval brains

Third instar larvae reared in ethanol show increased anti-NPF fluorescence in the axons of NPFergic neurons projecting to the central brain as well as the SEG. This is consistent with the known effects of sugar on NPF cell projections (Shen and Cai, 2001), and would be expected to increase foraging behavior for at least two reasons: first, the SEG contains both afferent and efferent nerves that regulate larval foraging behavior (Altman and Kien, 1987). Second, increased NPF signaling prolongs foraging and feeding behavior in third instar larvae (Wu et al., 2003). In addition, because ethanol-containing food is unpalatable (Kaun et al., 2011), and NPF signaling increases the willingness of larvae to ingest unpalatable food (Wu et al., 2005), this increase would be expected to increase the overall amount of food consumed.

It is also possible that NPF release is being inhibited in ethanol-reared animals, and the increased anti-NPF foci in the SEG reflects a loss of NPF neuron function rather than increased signaling in these animals. We think this explanation is less likely due to previous data demonstrating the NPF signaling is enhanced by rewarding substances, and that the activation of NPF neurons in flies is sufficient to mimic the effects of ethanol reward, suggesting that alcohol does not prevent the release of NPF in flies, but, rather, tends to enhance it (Shohat-Ophir et al., 2012).

NPF is expressed in both the brain and in enteroendocrine cells in the midgut of larvae as well as adult flies (Brown et al., 1999). Here, we focus on the expression of NPF in the larval brain, but it should be noted that our experiments would alter midgut NPF expression, and we have not investigated possible involvement of midgut cells in the regulation of hunger in ethanol-supplemented animals.

### NPF signaling is essential in the presence of ethanol

Loss of NPF signaling through a genetically null mutation in *npfr1* leads to death during early larval stages for most ethanol-reared animals. This was a surprising result, as, to our knowledge, there has been no lethality previously associated with loss of NPF signaling. In our experiments, *npfr1* is not required for survival under normal environmental conditions, as homozygosity for a null mutation in *npfr1* results in no significant change in survival compared to wildtype. Thus, we have identified an environmental condition under which *npfr1* is an essential gene.

Signaling by the Drosophila insulin-like peptides (DILPs) also affects food intake (Wu et al., 2005). Specifically, it is thought that DILPs signal to and inhibit the activity of NPFR1-expressing cells, such that, when animals are well-fed, DILP signaling leads to reduced feeding (and reduced acceptance of noxious foods). We have previously shown that DAE leads to a 75% reduction in expression of the Drosophila insulin receptor (InR) (McClure et al., 2011). Thus, it is interesting to speculate that the combination of reduced insulin signal and increased NPF signal upon DAE would lead to increased acceptance of ethanol-tainted food. This hypothesis predicts that mutations leading to reduced insulin signaling should exert a protective effect in the presence of an NPFR mutation, and, conversely, that overexpression of DILPs should exacerbate the effects of an NPFR mutation. We are currently performing experiments to test these predictions.

**Figure 5:**
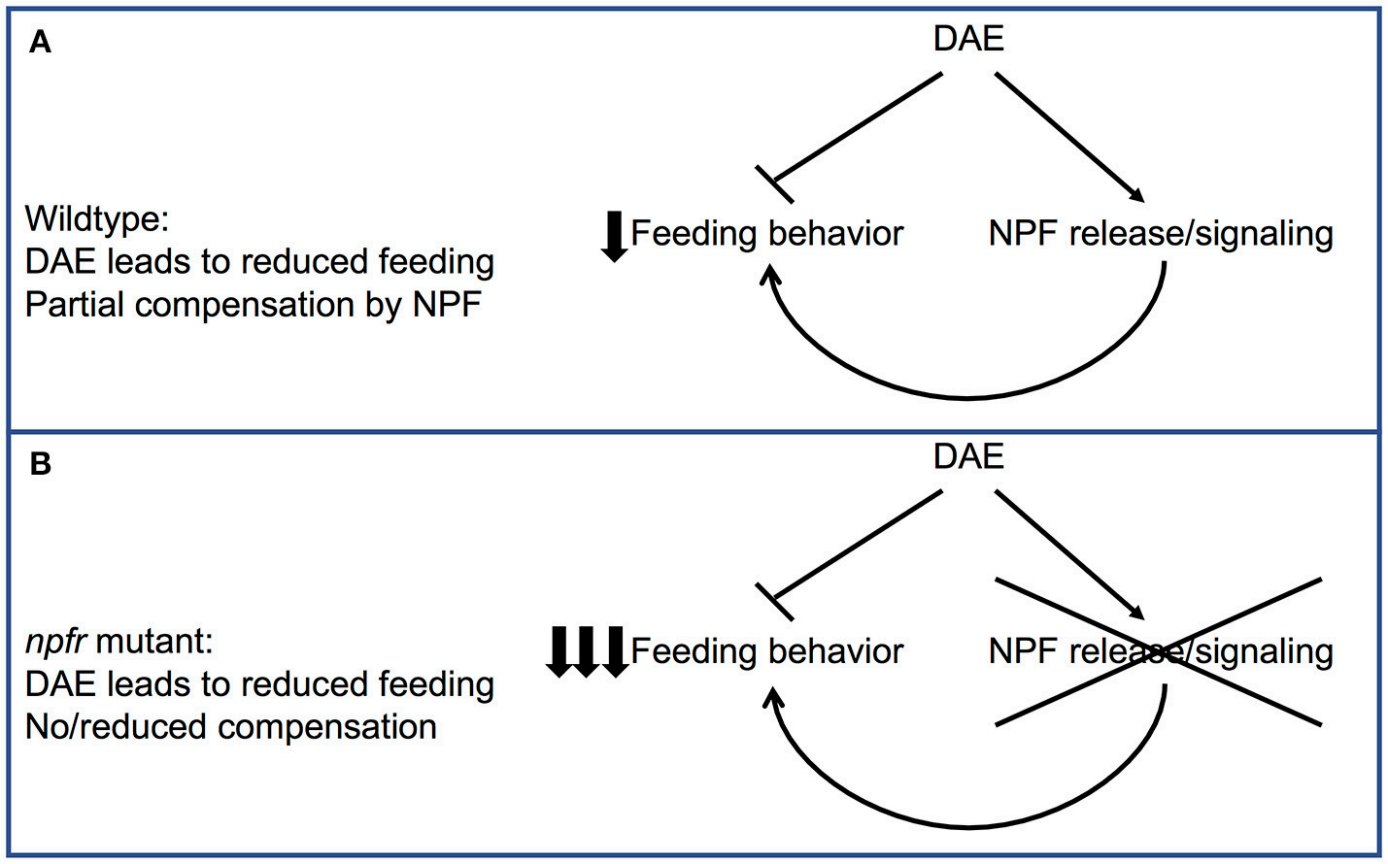
Model for the effect of loss of NPF signaling on feeding in ethanol-supplemented larvae. **(A)** In wildtype animals, eating food containing ethanol leads to a decrease in feeding behavior as well as an increase in NPF release/signal transduction. This increase in NPF release would be expected to partially compensate for the effect of ethanol on feeding. **(B)** In *npfr* mutant animals, this compensatory upregulation in feeding behavior cannot occur, leading to a much larger reduction in feeding.

Taken together, these data suggest a model in which DAE causes increased NPF signaling, as well as abnormal feeding through as-yet unidentified molecular targets (Figure 5A). In this model, increased NPF expression in ethanol-supplemented animals has evolved in part as a compensatory or protective mechanism, in which NPF expression offsets to some degree the reduced food intake caused by DAE. When flies lack the ability to increase NPF signaling due to a mutation in *npfr1*, the combination of reduced feeding due to ethanol exposure and loss of a reward pathway that would serve to drive increased foraging and food intake may result in larvae that eat too little to sustain growth and development (Figure 5B).

It should be noted that, though feeding was significantly reduced in *npfr1* mutant animals reared in ethanol, more than half of these animals have nevertheless consumed food by the end of a 45-min observation period (Figure 3B). It is possible that, despite this, the volume of food consumed by these animals is insufficient to sustain growth, leading to the lethality associated with the combination of ethanol and reduced NPF signaling. It is also possible that NPF signaling is affecting survival through an as-yet-unidentified mechanism that impacts survival. One possibility is that ethanol metabolism is altered in *npfr1* mutant animals, such that ethanol is toxic to these mutants at the concentrations described here. We think that possibility is unlikely, given that we see no sedative effects on locomotion in *npfr1* mutant animals when reared in food containing 7% ethanol (Supplemental Figure 1).

In conclusion, we have shown that flies reared in ethanol display feeding changes consistent with the effects of FASD in mammals, and that DAE induces NPF signaling to regions of the central nervous system that drive foraging and food intake, and, finally, that loss of this compensatory mechanism results in additional reductions in food intake and a very high rate of developmental lethality, suggesting that the cause of death for *npfr1/npfr1* mutant larvae may be “voluntary” starvation. Our data also suggest that NPY receptor agonists may have potential for treating feeding difficulties associated with FASD.

## Author contributions

AG and RF: Conceived and designed the experimental plan; AG, HG, and BU: Performed the experiments; AG, HG, BU, and RF: Analyzed the data; AG and RF: Drafted the manuscript.

### Conflict of interest statement

The authors declare that the research was conducted in the absence of any commercial or financial relationships that could be construed as a potential conflict of interest.

## Supplementary material

Supplemental Figure 1 Ethanol-rearing does not cause sedation in first instar larvae. Distance traveled in in ethanol-free medium in 3 min by first instar larvae. Distance traveled was similar for wildtype larvae reared in control food, *NPFR1/NPFR1* larvae reared in control food, and *NPFR1/NPFR1* larvae reared in ethanol-containing food. Wildtype larvae reared in ethanol-containing food moved more, on average, than all other conditions. Locomotion data were not normally distributed. Statistics were performed on log-transformed data. (*N* = 10 for all conditions, *P* = 0.82 for the effect of genotype, *P* = 0.025 for the effect of ethanol, *P* = 0.079 for the interactions between ethanol and genotype.) Center lines show the back-transformed sample mean; box limits indicate the 25th and 75th percentiles as determined by R software; whiskers extend 1.5 times the interquartile range from the 25th and 75th percentiles.
