# Supplementary figures and images for: Developmental Ethanol Exposure Causes Reduced Feeding and Reveals a Critical Role for Neuropeptide F in Survival
Source: Front Physiol. 2018 Mar 22;9:237. doi: 10.3389/fphys.2018.00237 (PMC5875382; doi:10.3389/fphys.2018.00237)

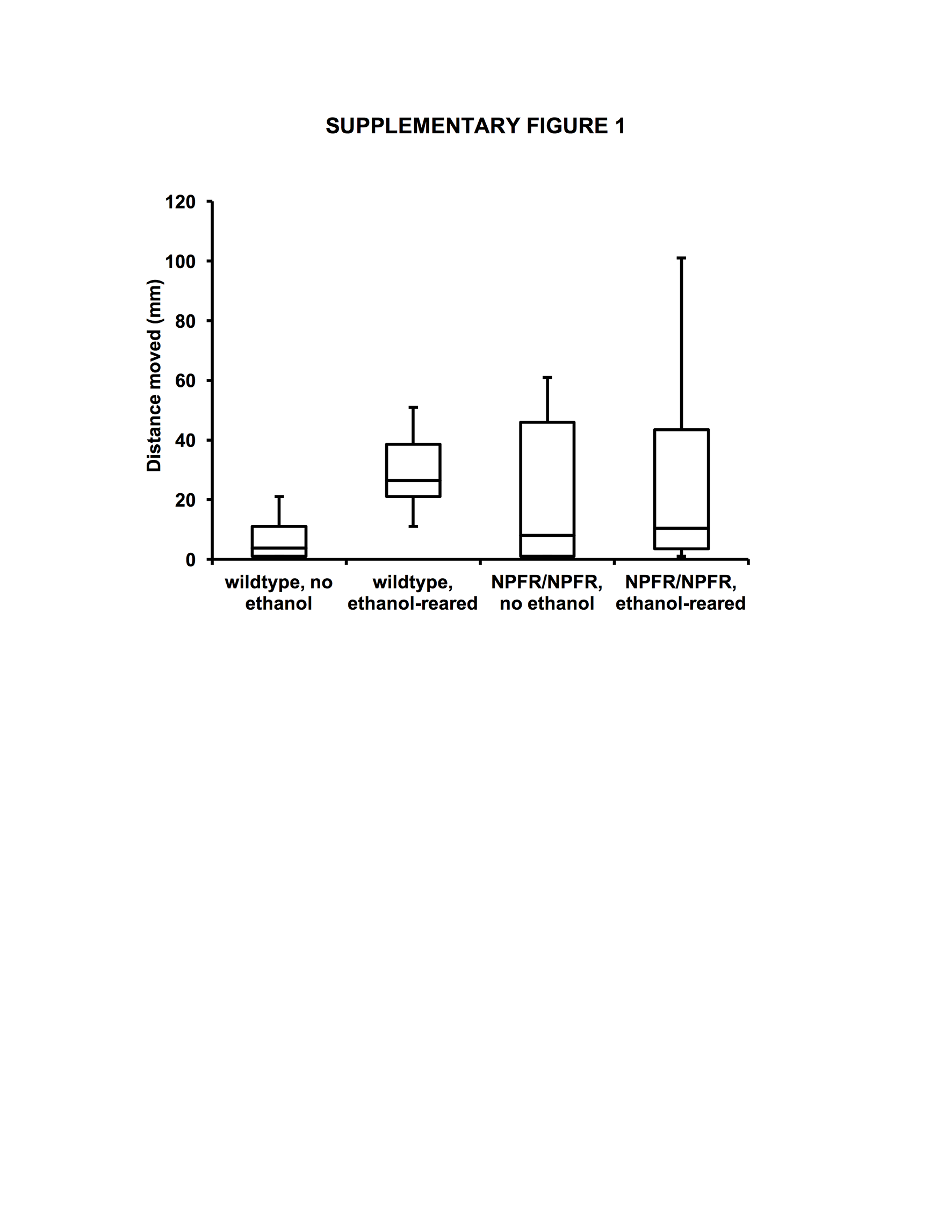

Supplement: Supplemental Figure 1 — Ethanol-rearing does not cause sedation in first instar larvae. Distance traveled in in ethanol-free medium in 3 min by first instar larvae. Distance traveled was similar for wildtype larvae reared in control food, NPFR1/NPFR1 larvae reared in control food, and NPFR1/NPFR1 larvae reared in ethanol-containing food. Wildtype larvae reared in ethanol-containing food moved more, on average, than all other conditions. Locomotion data were not normally distributed. Statistics were performed on log-transformed data. (N = 10 for all conditions, P = 0.82 for the effect of genotype, P = 0.025 for the effect of ethanol, P = 0.079 for the interactions between ethanol and genotype.) Center lines show the back-transformed sample mean; box limits indicate the 25th and 75th percentiles as determined by R software; whiskers extend 1.5 times the interquartile range from the 25th and 75th percentiles. [file Image1.TIFF]
